# Movement Discordance between Healthy and Non-Healthy US Adults

**DOI:** 10.1371/journal.pone.0150325

**Published:** 2016-02-26

**Authors:** Ann M. Swartz, Young Cho, Whitney A. Welch, Scott J. Strath

**Affiliations:** 1 Department of Kinesiology, University of Wisconsin-Milwaukee, Milwaukee, WI, United States of America; 2 Center for Aging and Translational Research, University of Wisconsin-Milwaukee, Milwaukee, WI, United States of America; 3 Zilber School of Public Health, University of Wisconsin-Milwaukee, Milwaukee, WI, United States of America; Universidad Europea de Madrid, SPAIN

## Abstract

**Introduction:**

Physical activity is known to significantly impact cardiometabolic health. Accelerometer data, as a measure of physical activity, can be used to objectively identify a disparity in movement (movement discordance) between healthy and unhealthy adults. The purpose of this study was to examine the M*ovement Discordance* between healthy and unhealthy adults in a large US population sample.

**Methods:**

Demographic, health and accelerometer data from the National Health and Nutrition Examination Study (NHANES) 2003–2004 and 2005–2006 cohorts were used for this study. Participants were classified as either having a “normal” or “abnormal” value for each cardiometabolic health parameter examined, based on published criteria. Linear regression analyses were performed to determine significance of each abnormal health parameter (risk factor) in its unique effect on the accelerometer counts, controlling for age and gender. Average accelerometer counts per minute (cpm) by gender and age categories were estimated separately for the groups of normal and abnormal cardiometabolic risk.

**Results:**

Average cpm for those with healthy levels of each individual cardiometabolic health parameter range from 296 cpm (for C reactive protein) to 337 cpm (for waist circumference), while average cpm for those with abnormal levels of each individual cardiometabolic health parameter range from 216 cpm (for insulin) to 291 cpm (for LDL-cholesterol). After controlling for age and gender, waist circumference, HbA1c, Insulin, Homocysteine, and HDL-Cholesterol were the cardiometabolic health parameters that showed significant, unique and independent effects on cpm. Overall, individuals who have abnormal values for all significant cardiometabolic health parameters (“unhealthy”) averaged 267 cpm (SE = 15 cpm), while the healthy sample of this study averaged 428 cpm (SE = 10 cpm). The difference in cpm between the unhealthy and healthy groups is similar between males and females. Further, for both males and females, the cpm gap between unhealthy and healthy is largest in the 30s (males: 183 cpm; females 144 cpm) and lessens as age increases, with the lowest gap seen in those 80+ years (males, 81 cpm; females, 85 cpm).

**Conclusion:**

This *Movement Discordance* between healthy and unhealthy adults represents a gap in movement that needs to be closed to improve the health of individuals with, or at risk for cardiometabolic disease.

## Introduction

Accelerometer-based motion sensors have become a widely used method to assess physical activity. Researchers have used various methods to interpret the accelerations recorded by the device, with the use of cut-points being the most widely employed. The cut-point method of interpreting accelerometer data gained quick popularity because it was easy to use and easy to interpret. Further, this method allowed researchers to determine whether individuals were accumulating sufficient moderate-to vigorous physical activity to meet national physical activity recommendations. However, as many have communicated [1,2], there are a number of concerns when using the cut-point method of interpreting accelerometer data. First, the ability to compare results between studies applying different cut points become difficult. Second, the cut points are only valid for the specific accelerometer in question and the populations and activities on which they have been developed, resulting in no universal cut-point method for assessing physical activity behavior.

Recently there has been interest in the use of total activity counts (TACs) to interpret accelerometer data. Based on the NHANES dataset, researchers have shown that TACs tend to decrease with age; males tend to have higher TACs than females, with the difference between genders decreasing with age [3,4]. Wolff et al reported that, for the NHANES 2003–2006 sample, males (all ages) in the 50^th^ percentile accumulated 288,140 TAC/day and females (all ages) in the 50^th^ percentile accumulated 235,741 TAC/day [5]. Males aged 20, 50, and 80 years in the 50^th^ percentile accumulated 345,920; 294,720; and 129,282 TAC/day, while females aged 20, 50, and 80 years in the 50^th^ percentile accumulated 259,903; 241,915; and 117,549 TAC/day, respectively. Finally, TAC has been shown to be more strongly associated with biomarkers of cardiometabolic health than moderate to vigorous physical activity accumulated in bouts of 10 minutes or longer, suggesting the importance of total daily activity to health [6]. Proponents of TACs suggest that this method of reporting accelerometer output provides information on the whole day of activity and includes both the health enhancing and health detracting behaviors because each accelerometer count is reflective of the intensity of the activity [1,5]. However, these values do not account for total daily wear time, and therefore may reflect 12 hours of activity for one individual and 16 hours of activity for another.

To address total daily wear time, others have used cpm for depicting activity patterns across age and gender. Troiano [4] reported mean activity counts per minute (cpm) from the 2003–2004 NHANES dataset. Accelerometry cpm ranged from 444.2 ± 13.4 cpm for 30–39 year old males to 169.8 ± 3.0 cpm for 70+ year old females. In general cpm remained relatively stable from age 20–39 years, after which a continual reduction was shown as age categories increased, and cpm were shown to be higher for males than females throughout adulthood. Finally, Everson [7] provided cpm profiles of individuals with varying levels of PA. The most active group averaged 852.4 cpm and the least active group averaged 135.4 cpm. Further, recent studies have reported cpm for those with disease. Individuals with angina (198 ±10 cpm), coronary heart disease (189 ±8 cpm), congestive heart failure (172 ±10 cpm), and myocardial infarction (197±9 cpm) had significantly lower cpm than the referent group without disease (245 ±7 cpm), however, stroke survivors (190 ±22 cpm) did not significantly differ from their referent group who did not have a stroke (227 ±22 cpm) [8,9].

Given the relationship between physical activity and chronic acquired diseases, it is important to explore differences in cpm between those who are healthy and those who have chronic acquired diseases. While researchers are beginning to use cpm more frequently, and provide normative values based upon demographic characteristics, there remains a paucity of data exploring TAC or cpm levels denoting thresholds relative to a full array of cardiometabolc risk factors. This study extends previous work by examining cpm for those with positive and negative risk factors by age and gender to identify a disparity in movement (Movement Discordance) between healthy and unhealthy adults. This Movement Discordance represents a gap in movement that needs to be closed to improve the health of individuals with chronic disease or at risk for chronic disease. Examining cpm to determine the Movement Discordance is a simple method that takes into account all the elements of movement and lack of movement (sedentary, light-, moderate- and vigorous-intensity physical activity), and presents a simple metric that supports the Move for Health message.

Therefore, the purpose of this study was to examine the Movement Discordance between healthy and unhealthy adults based on NHANES data. Specifically, this study aimed to determine the cpm values associated with a healthy individual, and those cpm values associated with elevated risk for cardiometabolic disease by age and gender.

## Methods

### Participants

Data from the National Health and Nutrition Examination Study (NHANES) 2003–2004 and 2005–2006 cohorts were used for this study. Data included the non-institutionalized, in-person home interview which collected demographic, socioeconomic, health-related information as well as the data collected during the mobile examination center visit (MEC; medical and laboratory measures). Inclusion criteria for these analysis consisted of ambulatory adults aged 20 years or older, valid accelerometer data (10h/d of accelerometer wear for at least 4 d) [4], and valid measures of the health variables of interest.

Participants were classified as either having a “normal” or “abnormal” value for each health parameter based on published criteria. Normal systolic blood pressure (SBP; mmHg) was defined as <120 and normal diastolic blood pressure (DBP; mmHg) was defined as <80 [10]. Normal body mass index (BMI; kg/m^2^) was defined as <25; Normal waist circumference was defined as Men< = 102, Females< = 88[11]. Normal total cholesterol (mg/dL) <200; Normal high density lipoprotein cholesterol (HDL-C; mg/dL) was defined as >40; Normal low density lipoprotein cholesterol (LDL-C; mg/dL) was defined as <100; Normal triglycerides (trig; mg/dL) was defined as <150 [12]. Normal HbA1c (%) was defined as <5.7; Normal fasting glucose (mg/dL) was defined as <100; Normal fasting insulin was defined as < 25 mIU/L (< 174 pmol/L) [13]. Normal C reactive protein was defined as 1.0–3.0 mg/L; Normal Plasma homocysteine was defined as Age 0–30 years: 4.6–8.1 μmol/L, Age 30–59 years: 6.3–11.2 μmol/L, (males); 4-5-7.9 μmol/L (females), Age >59 years: 5.8–11.9 μmol/L [14,15].

### Study Design & Measures

Details on the NHANES procedures can be found on the CDC’s website[16].

As part of the NHANES MEC data collection, participants were asked to wear an Actigraph 7164 accelerometer on their right hip during all waking hours for seven consecutive days, and only to remove it while swimming, bathing, or while submerged in water. Data was collected in 60- second epochs. Vertical accelerations were filtered, full-wave rectified, and integrated over time to produce a “count” representative of the intensity of the activity being performed [17].

Non-wear time was defined as 60 or more consecutive minutes of zero counts, allowing up to 2 consecutive minutes with limited movement (<100 cts/min) within the period [4].

A valid day was defined as 10 or more hours of wear, where daily wear time was calculated by subtracting non-wear time from 24 hours[4]. A valid week was defined as 4 or more days [4]. The primary output from the accelerometers was cpm of the vertical axis.

### Statistical Analysis

All analyses were conducted using Stata 13 with the survey data analysis module to account for the complex sampling design NHANES employs and the sample weights calculated according to the NHANES analytical guidelines [18]. The outcome variable was normalized by transformation into cube rooted average cpm. Descriptive analyses were conducted to estimate differences between groups of normal and abnormal status of each cardiometabolic risk factors in the level of activity cpm. Second, linear regression analyses were performed to determine significance of each risk factor in its unique effect on the activity counts, controlling for age and gender. Third, average activity cpm by gender and age categories were estimated separately for the groups of normal and abnormal cardiometabolic risk statuses. Analyses were based on a sample of individuals who met the aforementioned inclusion criteria.

## Results

There were 6091 individuals from the NHANES 2003–2004 and 2005–2006 samples who met our inclusion criteria (20+ years of age, valid accelerometer wear time with one or more of the health variables measured) for this analysis (Table 1). However, sample sizes for each specific health variable were lower, based on inclusion/exclusion criteria (Table 2). Overall, approximately half of the sample was female, the majority of the sample was Non-Hispanic White, and over half the sample had a least some college and/or an Associate degree.

**Table 1:** Descriptive Statistics of Sample (N = 6091).

	Mean (or %)
Age (yr)	48.32 (SE = .45)
Gender (% female)	51.7%
Race/ethnicity (%)	
Non-Hispanic White	74.0%
Non-Hispanic Black	9.8%
Mexican American	11.0%
Other	5.2%
Education	
< High School	15.8%
High School degree/ GED	25.5%
Some College and/or Associate degree	32.0%
College degree or advance degree	26.7%

**Table 2:** Proportion of sample* with normal or abnormal cardiometabolic health values.

		Normal Value		Abnormal Value	
	Total N	n	%	n	%
Systolic blood pressure	5913	2216	41.5%	3697	58.5%
Diastolic blood pressure	5913	3394	59.7%	2519	40.3%
BMI	6042	1860	32.8%	4182	67.2%
Waist circumference	5920	2650	46.7%	3270	53.3%
Total cholesterol	5930	2443	42.9%	3487	57.2%
HDL-Cholesterol	2997	2144	73.0%	853	27.0%
LDL-Cholesterol	2705	692	25.9%	2013	74.1%
Triglycerides	2785	1546	59.2%	1239	40.8%
Glucose	2995	1567	57.0%	1428	43.0%
HbA1c	5926	4165	77.1%	1761	22.9%
Insulin	2972	2476	86.5%	496	13.5%
C reactive protein	5897	5820	98.8%	77	1.2%
Homocysteine	5894	4057	70.4%	1837	29.6%

Note.

*Among those 20+ years old with valid day and time of wear. Normal systolic blood pressure (SBP; mmHg)<120; Normal diastolic blood pressure (DBP; mmHg) <80; Normal body mass index (BMI; kg/m^2^)<25; Normal waist circumference Males< = 102, Females< = 88; Normal total cholesterol (mg/dL) <200; Normal high density lipoprotein cholesterol (HDL-C; mg/dL) <40; Normal low density lipoprotein cholesterol (LDL-C; mg/dL) <100; Normal triglycerides (trig; mg/dL) <150; Normal HbA1c (%)<5.7; Normal fasting glucose (mg/dL)<100; Normal fasting insulin < 25 mIU/L (< 174 pmol/L); Normal C reactive protein 1.0–3.0 mg/L; Normal Plasma homocysteine Age 0–30 years: 4.6–8.1 μmol/L, Age 30–59 years: 6.3–11.2 μmol/L, (males); 4-5-7.9 μmol/L (females), Age >59 years: 5.8–11.9 μmol/L.

Of the sample included in this analysis, over 40% of the sample had elevated systolic blood pressure, diastolic blood pressure, BMI, waist circumference, total cholesterol, LDL-C, triglycerides and glucose (Table 2). Less than one-third of the sample had abnormal levels of HDL-C, HbA1c, fasting insulin, C-reactive protein, and homocysteine.

Table 3 shows the mean cpm for individuals who have normal levels versus abnormal levels of each individual cardiometabolic risk factor. Average cpm for those with healthy levels of each individual cardiometabolic risk factor range from 296 cpm to 337 cpm, while average cpm for those with abnormal levels of each individual cardiometabolic risk factor range from 216 cpm to 291 cpm. Significant differences between cpm for those with normal and abnormal levels of each individual cardiometabolic risk are present for all risk factors, with differences ranging from as little as 26 cpm to as large as 88 cpm.

**Table 3:** Counts per Minute of Sample by Normal vs. Abnormal Cardiometabolic Risk Factors.

Cardiometabolic risk factors	Normal		Abnormal		Difference Test	
	Mean*	SE	Mean*	SE	t	p
Systolic blood pressure	332	3	270	4	13.07	0.000
Diastolic blood pressure	322	3	257	5	11.63	0.000
BMI	316	4	286	4	5.62	0.000
Waist circumference	337	3	264	3	19.07	0.000
Total cholesterol	317	4	279	3	7.99	0.000
HDL-Cholesterol	310	5	257	8	6.47	0.000
LDL-cholesterol	314	8	291	6	2.47	0.019
Triglycerides	320	4	262	8	7.00	0.000
Glucose	319	5	256	6	8.72	0.000
HbA1c	313	3	239	5	13.22	0.000
Insulin	304	4	216	8	10.40	0.000
C reactive protein	296	3	238	15	3.87	0.001
Homocysteine	303	3	277	4	5.35	0.000

Note.

*back-transformed from cube rooted cpm.

The overall model to predict cube rooted cpm from cardiometabolic factors, controlling for age and gender, was significant (R^2^ = .39, p < .0001). Based on the results of the linear regression, five risk factors showed significant, unique and independent effects on cube rooted cpm after controlling for gender and age: waist circumference (b = 0.261, SE = 0.053, t = 4.95, p<0.001), HbA1c (b = 0.167, SE = 0.037, t = 4.48, p<0.001), Insulin (b = 0.307, SE = 0.090, t = 3.42, p = 0.004), Homocysteine (b = 0.223, SE = 0.055, t = 4.06, p = 0.001), and HDL-Cholesterol (b = 0.178, SE = 0.082, t = 2.170, p = 0.047), where the beta coefficient of each risk factor indicates the difference between normal and abnormal condition in the cube-rooted cpm. Together, these five risk factors accounted for 14% of the variance in the activity cpm. These data suggest the importance of metabolic (waist circumference, insulin, HbA1c) and cardiovascular health (homocysteine, HDL-C) to the activity level of the individual.

**Fig 1:**
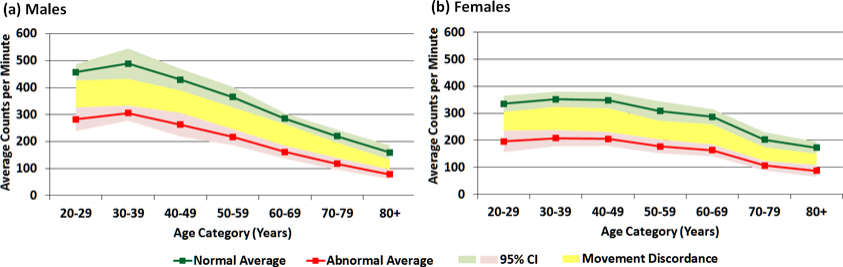
Movement Discordance Gap between healthy individuals and those with cardiometabolic disease risk factors (a) by age for males and (b) by age for females.

Because age and gender were unique and significant contributors to cpm in the model, cpm were explored by age and gender for those with and without cardiometabolic risk factors. Table 4 and Fig 1 show estimated mean cpm for males (Fig 1A) and females (Fig 1B) by age category who have normal values and who have abnormal values for those 5 cardiometabolic risk factors identified in the linear regression model. Overall, the healthy sample of this study averaged 428 cpm (SE = 10 cpm), with the highest cpm occurring for males and females in their 30s. The difference in cpm between healthy males and females is the largest in the fourth decade of life (ages 30–39 years; d = 137, p = < .001) and decreases with age. In those healthy individuals 60 years and older, there is little difference in cpm between males and females. For males, cpm increased from 20–29 to 30–39y, and then declined thereafter, with those healthy males aged 80+ years averaging 329 cpm less than healthy males aged 30–39 years. For healthy females, cpm increased slightly from 20–29 to 30–39 years, then remain fairly constant from 30–39 to 40–49 years after which cpm decreased with increasing age. Healthy females show a similar magnitude of decrease in cpm to males, with 300 fewer cpm, on average, at age 80+ years compared with 30–39 years.

**Table 4:** Estimated Means of Activity Counts per Minute for healthy adults and adults with cardiometabolic disease risk factors^#^ by age and gender (N = 1426*).

	Normal Cardiometabolic Risk Factors		Abnormal Cardiometabolic Risk Factors	
	Mean	SE	Mean	SE
**Male**				
age 20–29	457	15	283	22
age 30–39	489	28	306	14
age 40–49	430	20	263	22
age 50–59	365	19	217	15
age 60–69	285	10	161	12
age 70–79	220	12	118	11
age 80+	160	13	79	9
**Female**				
age 20–29	335	15	196	20
age 30–39	352	14	208	15
age 40–49	348	15	205	13
age 50–59	308	18	177	13
age 60–69	287	14	163	11
age 70–79	202	14	106	9
age 80+	172	11	87	11

Note

*Overall estimation; gender and age adjusted; No Cardiometabolic Risk Factors (all 5 risk factors within normal limits)

^#^ Cardiometabolic Risk Factors include those significant contributors to the model: waist circumference, HbA1c, insulin, homocysteine, and HDL-cholesterol

Overall, individuals who have abnormal values for all 5 cardiometabolic risk factors (“unhealthy”) averaged 267 cpm (SE = 15 cpm), with the highest cpm occurring for males and females in their 30s (Table 4). Unhealthy males tended to have higher cpm values than unhealthy females, with the largest gender difference in cpm seen in the 30–39 year age category (98 cpm). This gender difference in cpm decreases with age. In those 60 years and older with cardiometabolic risk factors, there is little difference in cpm between males and females. For unhealthy males, cpm increases from the age group of 20–29 to 30–39, and then declines thereafter, with those unhealthy males aged 80+ years averaging 329 cpm less than males aged 30–39 years. For unhealthy females, cpm increases slightly from 20–29 years to 30–39 years, then remain fairly constant from 30–39 to 40–49 years, and thereafter cpm decrease with increasing age. Unhealthy females show a similar magnitude of decrease in cpm to males, with 300 fewer cpm, on average, at age 80+ years compared with 30–39 years.

The discordance in cpm between healthy individuals and those with cardiovascular risk factors is shown in Fig 1. The difference in cpm between those with cardiometabolic risk factors and those without is similar between males and females (Fig 1A & 1B), however, the cpm difference between healthy individuals and individuals with all 5 of the cardiometabolic disease risk factors ranges from 183 cpm (30–39y) to 81 cpm (80+ years) in males (Fig 1A) and 144 cpm (30–39y) to 85 cpm (80+ years) in females (Fig 1B). For both males and females, the gap is lessened as age increases.

## Discussion

Research has begun to explore TAC or cpm as a metric of activity, and provided normative cpm based on demographic characteristics, or a single disease. However, there is a knowledge gap in the current literature pertaining to TAC or cpm thresholds relative to a full array of cardiometabolc risk factors. This study is the first to report a *Movement Discordance* in cpm between individuals with and without cardiometabolic risk factors using a nationally representative sample. These differences represent a physical activity gap between healthy and unhealthy or at-risk individuals, with average cpm of 428 ± 10 cpm for those with healthy levels of individual cardiometabolic risk factors to 267 ± 15 cpm for those with abnormal levels of individual cardiometabolic risk factors. When extrapolating these values out for a full 14 hour day, the healthy individual would accumulate 359,520 total activity counts, and the individual with cardiometabolic risk factors would accumulate 224,280 total activity counts. When comparing these values to the population-referenced percentiles for total activity counts [5] for a 48 year old (the mean age of this sample), this would place a female between the 75^th^ and 90^th^ percentile and between the 25^th^ and 50^th^ percentile, respectively; while a male would be placed between the 50^th^ and 75^th^ percentiles and just below the 25^th^ percentile, respectively. Further, these mean values are higher than those reported for individuals with diagnosed cardiovascular disease [8,9]. This discordance represents a goal for individuals to strive towards- those with disease should work towards increasing their cpm up to the level of those without disease through an increase in physical activity and/or a decrease in sedentary behavior. This discordance represents a public health opportunity- an activity goal in which all movement and time spent in sedentary behavior is accounted.

When examining each individual risk factor, differences in cpm between those with and without the risk factor range from as little as 26 cpm for homocysteine to as large as 88 cpm for insulin, with the largest gaps, in general, seen in risk factors associated with glucose homeostatis and the smallest gaps seen in risk factors associated with cholesterol. These differences in cpm for risk factors are not insurmountable for an unhealthy individual to achieve. For example, if an individual sits for 10.5 h/d (average cpm = 50), engages in 5h 15min/day of light intensity physical activity (average cpm = 500) and engages in 15 min of moderate intensity physical activity (average cpm = 3000), this individual would average 244 cpm for the day. If this individual then replaced 30 minutes of sitting (50 cpm) with 30 minutes of moderate intensity activity (3000 cpm), their average cpm would be 336, a difference of 92 cpm. Alternatively, if this individual did not change the amount of time in any intensity of physical activity or the amount of time they sat, but increased the average cpm in light intensity from 500 to 1000 cpm, this would result in an increase in the average cpm for the day to 408 cpm, a difference of 164 cpm. Therefore, these data support the notion that small changes in physical activity or sedentary behavior can impact the health of an individual with disease.

Previous researchers have reported cpm by age and gender [4–7,19], with decreases in moderate- to vigorous physical activity (MVPA) contributing to the decrease in total activity counts, while light intensity physical activity (LPA) remains stable [4,5,20]. The data in this study support previous studies showing a decrease in cpm with age, and a gender influence with males generally having higher cpm than females until the seventh decade of life. After approximately 75 years of age, females accumulate higher cpm than males. These data extend this previous research to examine the influence of cardiometabolic risk factors on cpm, while accounting for age and gender. Results of this study show a strong influence of risk factors on activity level, even after accounting for age and gender. However, it should be noted that this movement gap gets smaller as age increases and the model presented in this paper shows that the cardiometabolic risk factors, age and gender only account for 40% of the variance in cpm, suggesting that there are other factors not explored in this paper that have a strong influence on physical activity level in adults.

Scientists have applied various modeling techniques to try to translate cpm, a measure of movement, into an indicator of physiologic response (kcals, METS), or into meaningful measures of movement/physiological response (time spent in various intensities of movement- MVPA). However, these secondary or tertiary data reduction techniques are only valid and reliable when applied to similar populations and using similar equipment and placement of equipment as the original development. Therefore, this study builds upon the work of Wolff and colleagues [20,21] and extends that work to examine the unique effect of disease on cpm, and provides cpm targets for individuals to aspire. A cpm goal allows the individual to achieve that goal by either engaging in more physical activity and/or by sitting less. If a sedentary individual is given a cpm goal, they may, at first, achieve that goal by sitting less and engaging in more LPA. As they become more comfortable with physical activity and begin to form habits around physical activity, they can start to engage in more intense physical activity to achieve that goal, and ultimately achieve the national physical activity recommendations.

This study is strengthened by the nationally representative sample and large sample size. Further, measurement of physical activity through objective methods and measurement of cardiometabolic risk factors provide concrete values on which to base these analyses. Finally, the use of first order data (cpm) removes any error that would be introduced and possibly magnified with the translation of data into second or third order data. Despite these strengths, limitations remain. The use of a single hip worn accelerometer may not record all movement, especially upper body work and movement with an external load. However, walking is the most commonly performed activity in the US [21], therefore a hip worn accelerometer should provide a good indication of overall activity level. Future studies should explore more deeply the patterns of physical activity and cpm within day and throughout the week to identify movement patterns and opportunities for intervention.

This study introduces a new approach to examine the relationship between movement and disease, providing a clear movement discordance between healthy adults and adults with disease. A clear movement gap, such as this, provides an opportunity for physicians, clinical exercise physiologists, and public health officials to use concrete physical activity goals to increase the physical activity levels and decrease sedentary time of those with whom they work in order to improve their cardiometabolic health.

## References

[1] Bassett DR, Troiano RP, McClain JJ, Wolff DL (2015) Accelerometer-based physical activity: total volume per day and standardized measures. Med Sci Sports Exerc 47: 833–838. 10.1249/MSS.0000000000000468 25102292

[2] Strath SJ, Kaminsky LA, Ainsworth BE, Ekelund U, Freedson PS, et al (2013) Guide to the assessment of physical activity: Clinical and research applications: a scientific statement from the American Heart Association. Circulation 128: 2259–2279. 10.1161/01.cir.0000435708.67487.da 24126387

[3] Wolff-Hughes DL, Fitzhugh EC, Bassett DR, Churilla JR (2015) Waist-Worn Actigraphy: Population-Referenced Percentiles for Total Activity Counts in U.S. Adults. J Phys Act Health 12: 447–453. 10.1123/jpah.2013-0464 24905055

[4] Troiano RP, Berrigan D, Dodd KW, Masse LC, Tilert T, et al (2008) Physical activity in the United States measured by accelerometer. Med Sci Sports Exerc 40: 181–188. 18091006 10.1249/mss.0b013e31815a51b3

[5] Wolff D, Fitzhugh E, Bassett D, Churilla J (2015) Waist-Worn Actigraphy: Population-Referenced Percentiles for Total Activity Counts in U.S. Adults. Journal of Physical Activity & Health 12: 447–453 24905055 10.1123/jpah.2013-0464

[6] Wolff-Hughes DL, Fitzhugh EC, Bassett DR, Churilla JR (2015) Total Activity Counts and Bouted Minutes of Moderate-to-Vigorous Physical Activity: Relationships With Cardiometabolic Biomarkers Using 2003–2006 NHANES. Journal of Physical Activity & Health 12: 694–700. 25109602 10.1123/jpah.2013-0463

[7] Evenson KR, Wen F, Metzger JS, Herring AH (2015) Physical activity and sedentary behavior patterns using accelerometry from a national sample of United States adults. Int J Behav Nutr Phys Act 12: 20 10.1186/s12966-015-0183-7 25889192 PMC4336769

[8] Evenson KR, Butler EN, Rosamond WD (2014) Prevalence of physical activity and sedentary behavior among adults with cardiovascular disease in the United States. J Cardiopulm Rehabil Prev 34: 406–419. 10.1097/HCR.0000000000000064 25068393 PMC4216242

[9] Butler EN, Evenson KR (2014) Prevalence of physical activity and sedentary behavior among stroke survivors in the United States. Top Stroke Rehabil 21: 246–255. 10.1310/tsr2103-246 24985392 PMC4146341

[10] James PA, Oparil S, Carter BL, Cushman WC, Dennison-Himmelfarb C, et al (2014) 2014 evidence-based guideline for the management of high blood pressure in adults: report from the panel members appointed to the Eighth Joint National Committee (JNC 8). JAMA 311: 507–520. 10.1001/jama.2013.284427 24352797

[11] (1998) Clinical Guidelines on the Identification, Evaluation, and Treatment of Overweight and Obesity in Adults—The Evidence Report. National Institutes of Health. Obes Res 6 Suppl 2: 51S–209S. 9813653

[12] National Cholesterol Education Program Expert Panel on Detection E, Treatment of High Blood Cholesterol in A (2002) Third Report of the National Cholesterol Education Program (NCEP) Expert Panel on Detection, Evaluation, and Treatment of High Blood Cholesterol in Adults (Adult Treatment Panel III) final report. Circulation 106: 3143–3421. 12485966

[13] American Diabetes A (2015) (2) Classification and diagnosis of diabetes. Diabetes Care 38 Suppl: S8–S16. 10.2337/dc15-S005 25537714

[14] Melmed S, Polonsky KS, Larsen PR, Kronenberg HM, editors (2011) Williams Textbook of Endocrinology. 12 ed. Philadelphia, PA: Elsevier Saunders.

[15] Ferri FF, editor (2012) Laboratory Tests and Interpretation of Results, Section IV. 1 ed: Elsevier Mosby.

[16] Centers for Disease Control and Prevention (2007) National Health and Nutrition Examination Survey (NHANES), Laboratory Procedures Manual. Atlanta, GA: Centers for Disease Control and Prevention.

[17] Chen KY, Bassett DR Jr. (2005) The technology of accelerometry-based activity monitors: current and future. Med Sci Sports Exerc 37: S490–500. 16294112 10.1249/01.mss.0000185571.49104.82

[18] Johnson CL, Paulose-Ram R, Ogden CL, Carroll MD, Kruszon-Moran D, et al (2013) National health and nutrition examination survey: analytic guidelines, 1999–2010. Vital Health Stat 2: 1–24. 25090154

[19] Copeland JL, Esliger DW (2009) Accelerometer assessment of physical activity in active, healthy older adults. J Aging Phys Act 17: 17–30. 19299836 10.1123/japa.17.1.17

[20] Hawkins MS, Storti KL, Richardson CR, King WC, Strath SJ, et al (2009) Objectively measured physical activity of USA adults by sex, age, and racial/ethnic groups: a cross-sectional study. Int J Behav Nutr Phys Act 6: 31 10.1186/1479-5868-6-31 19493347 PMC2701914

[21] Siegel PZ, Brackbill RM, Heath GW (1995) The epidemiology of walking for exercise: implications for promoting activity among sedentary groups. Am J Public Health 85: 706–710. 7733433 10.2105/ajph.85.5.706 PMC1615430

